# Hevin Plays a Pivotal Role in Corneal Wound Healing

**DOI:** 10.1371/journal.pone.0081544

**Published:** 2013-11-26

**Authors:** Shyam S. Chaurasia, Promoda R. Perera, Rebekah Poh, Rayne R. Lim, Tina T. Wong, Jodhbir S. Mehta

**Affiliations:** 1 Tissue Engineering and Stem Cell Group, Singapore Eye Research Institute, Singapore, Singapore; 2 Signature Research Program in Neuroscience and Behavioral Disorders, Duke-NUS Graduate Medical School, Singapore, Singapore; 3 Department of Ophthalmology, Yong Loo Lin School of Medicine, National University of Singapore, Singapore, Singapore; 4 Singapore National Eye Centre, Singapore, Singapore; 5 School of Material Science and Engineering, Nanyang Technological University, Singapore, Singapore; 6 Department of Clinical Sciences, Duke-NUS Graduate Medical School, Singapore, Singapore; University of Missouri-Columbia, United States of America

## Abstract

**Background:**

Hevin is a matricellular protein involved in tissue repair and remodeling via interaction with the surrounding extracellular matrix (ECM) proteins. In this study, we examined the functional role of hevin using a corneal stromal wound healing model achieved by an excimer laser-induced irregular phototherapeutic keratectomy (IrrPTK) in hevin-null (hevin^-/-^) mice. We also investigated the effects of exogenous supplementation of recombinant human hevin (rhHevin) to rescue the stromal cellular components damaged by the excimer laser.

**Methodology/Principal Findings:**

Wild type (WT) and *hevin* ^*-/-*^ mice were divided into three groups at 4 time points- 1, 2, 3 and 4 weeks. Group I served as naïve without any treatment. Group II received epithelial debridement and underwent IrrPTK using excimer laser. Group III received topical application of rhHevin after IrrPTK surgery for 3 days. Eyes were analyzed for corneal haze and matrix remodeling components using slit lamp biomicroscopy, in vivo confocal microscopy, light microscopy (LM), transmission electron microscopy (TEM), immunohistochemistry (IHC) and western blotting (WB). IHC showed upregulation of hevin in IrrPTK-injured WT mice. *Hevin* ^*-/-*^ mice developed corneal haze as early as 1-2 weeks post IrrPTK-treatment compared to the WT group, which peaked at 3-4 weeks. They also exhibited accumulation of inflammatory cells, fibrotic components of ECM proteins and vascularized corneas as seen by IHC and WB. LM and TEM showed activated keratocytes (myofibroblasts), inflammatory debris and vascular tissues in the stroma. Exogenous application of rhHevin for 3 days reinstated inflammatory index of the corneal stroma similar to WT mice.

**Conclusions/Significance:**

Hevin is transiently expressed in the IrrPTK-injured corneas and loss of hevin predisposes them to aberrant wound healing. *Hevin* ^*-/-*^ mice develop early corneal haze characterized by severe chronic inflammation and stromal fibrosis that can be rescued with exogenous administration of rhHevin. Thus, hevin plays a pivotal role in the corneal wound healing.

## Introduction

Corneal wound healing involves a complex series of interactions between infiltrating cells, cytokines and extracellular matrix (ECM) proteins [1,2]. It is regulated by a variety of growth factors (TGFβ, KGF, EGF, FGF, PDGFβ), cell migration, cell proliferation and matrix remodeling proteins [3,4]. The cellular and molecular events involving corneal wound healing have been extensively studied in the cornea. Damage to the corneal epithelium releases pro-inflammatory cytokines such as interleukin-1, transforming growth factor-β (TGFβ) and platelet-derived growth factor (PDGF) [5] to activate stromal keratocytes into myofibroblasts at the site of injury [6,7], thus resulting in wound contraction and reorganization of extracellular matrix in the corneal stroma [8-10]. Recently, we have shown that the cellular transdifferentiation of corneal keratocytes to myofibroblasts usually requires 3-4 weeks, with few intermittent precursor cells expressing vimentin and desmin, in addition to αSMA [11]. During the early phase of wound healing, these cells are usually cleared from the wounded areas by apoptosis [12,13]. Remodeling of the corneal architecture after injury requires ECM proteins. Corneal transparency has been shown to be directly affected by the arrangement of the collagen fibrils, and any excessive or disorganization of the matrix during the wound healing process can lead to corneal scarring, resulting in a reduction of visual acuity [14]. Reorganization of ECM is modulated by myofibroblasts, matrix degrading enzymes, and integrins. These components along with other structural and regulatory proteins facilitate and contribute to the restoration of an effective wound healing mechanism [15].

Matricellular proteins belong to a group of regulatory ECM proteins designated to play a multifunctional role in the cell-matrix interactions, cell proliferation, and are typically expressed in the cells undergoing wound repair and remodeling [16]. Hevin (also known as SC-1, MAST9, SPARC-like 1 and ECM2) is a matricellular protein, which is widely expressed in several cell types, e.g., brain neurons, heart, muscle cells, kidney cells and dermal fibroblasts [17], and shares approximately 60% structural identity to secreted protein acidic and rich in cysteine (SPARC) [18]. Hevin has been shown to be involved in the development and regeneration of the central nervous system via selective transport into cellular processes of Bergmann glial cells [20], muscle differentiation [21], and in lymphocyte transendothelial migration in the immune system [22]. Its importance in growth and development has been widely discussed [19,23-26] and is commonly associated with regulation of cell migration and modulation of ECM proteins [27,28]. Hevin binds to collagen I and regulates decorin and collagen fibrillogenesis during development and tissue modeling [28-30]. In hevin-null mice, dermal wound bed showed abnormal proteoglycan levels and irregular collagen matrix [29]. Given its extracellular Ca^2+^ binding domain, a unique characteristic feature of SPARC family of glycoproteins, hevin has also been shown to reverse focal adhesion formation, thereby inhibiting cell migration and proliferation [18,19]. Moreover, reduced hevin expression has been implicated in the metastasis of cancer cells via de-adhesion and also in anti-proliferative functions [31,32]. 

Localization and functional role of hevin in the eye has not been extensively studied. Hevin has been found to be expressed in the trabecular meshwork tissue but does not appear to play any critical role in intraocular pressure (IOP) regulation [33]. The expression and functional role of hevin in the cornea is not known yet. Hevin has been found to be involved in the inflammatory phase in skin wound healing where it recruits macrophage to initiate and propagate the formation of new tissue [34]. The involvement of hevin in the early stages of inflammation and its tendency to bind ECM proteins especially during tissue remodeling/wound healing [28,34] in the injury site suggests a major role of this protein in the repair mechanism. Understanding the participation of matricellular proteins during corneal injury using targeted deletion of a hevin null mouse model (*hevin* ^*-/-*^) may unveil their role in orchestrating interactions with inflammatory, angiogenesis, and fibrotic proteins for efficient wound healing – a prerequisite for reinstating a normal clear cornea. To explore this possibility, we used the irregular phototherapeutic keratectomy (IrrPTK) excimer injury model in *hevin* ^*-/-*^ mice to investigate the functional role of hevin in corneal inflammation and fibrosis. In addition, we also rescued these corneas with supplementation of human recombinant hevin (rhHevin) to compensate the loss of hevin during the reorganization of the corneal stroma matrix.

## Materials and Methods

### Ethics statement

All procedures in animals were performed in accordance with the ARVO Statement for the Use of Animals in Ophthalmic and Vision Research. Animal experimentation protocols were approved by SingHealth Experimental Medicine Centre (SEMC) IACUC committee. 

### Animals and Study design

Eight- to 10-week-old C57BL/6 (129SVE) Wild type (WT) and Hevin-null (*hevin* ^*-/-*^) mice were maintained in the SEMC animal facility in 12h light and 12h dark cycle with access to food and water ad libitum. Anesthesia was achieved by intraperitoneal injection of ketamine hydrochloride (30 mg/kg) and xylazine hydrochloride (5 mg/kg). In addition, 1% proparacaine hydrochloride (Alcon, Ft. Worth, TX, USA) was applied topically to each eye at the time of surgery. The mice were then treated with topical antibiotic post-surgery. Euthanasia was performed with an intravenous injection of 100mg/kg pentobarbital while the animal was under general anesthesia. 

**Figure 1:**
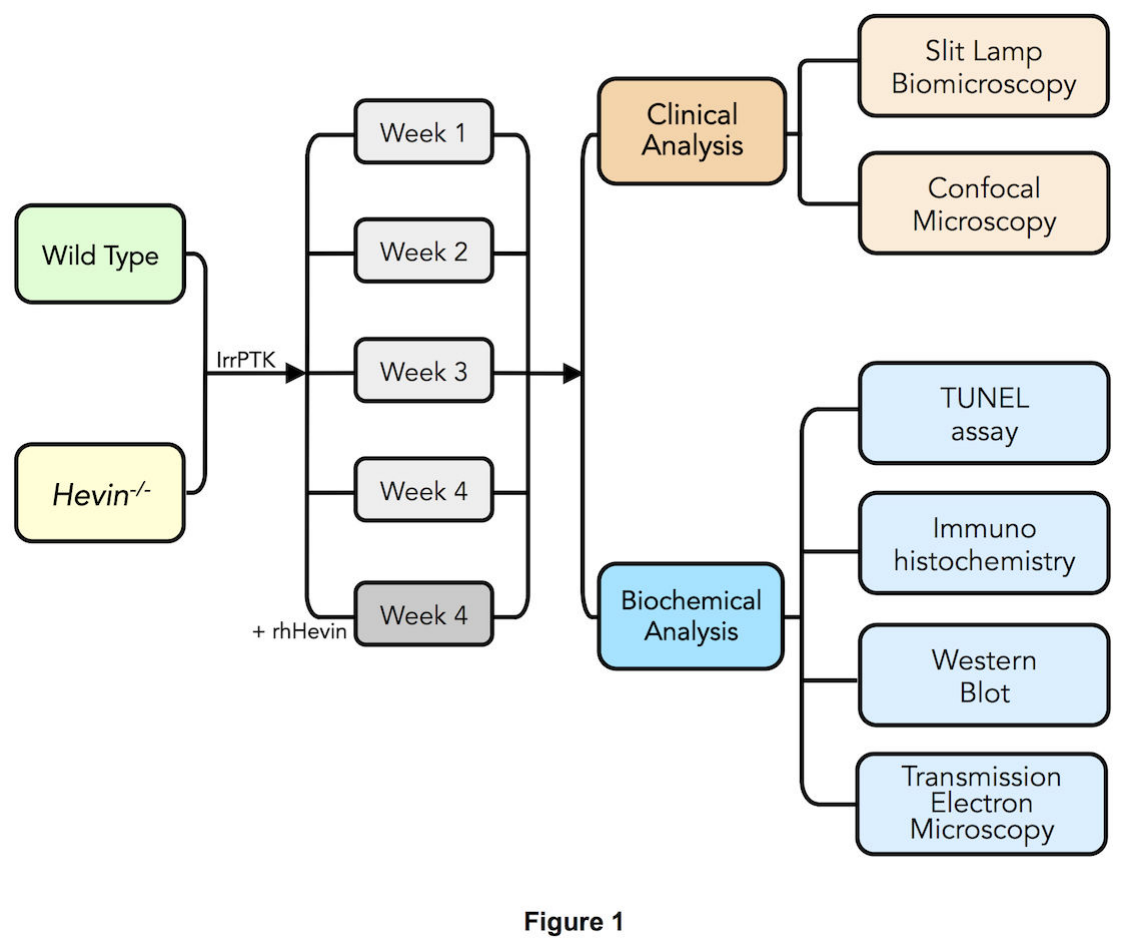
Flow diagram describing the experimental plan and data collection for the study. Eight-10 weeks old WT and *hevin^-/-^* mice were divided into 3 groups: (I) Naïve, (II) Irregular Phototherapeutic Keratectomy (IrrPTK), (III) IrrPTK + rhHevin. Group I without any treatment served as naïve control. Groups II and III underwent IrrPTK after epithelial debridement. rhHevin (10mg/Kg) was topically administered to Group III animals every 24 hours for 3 days until epithelial closure. Each group consists of 4 time points at 1, 2, 3, 4 weeks with 6-10 eyes at each time point. Slit lamp and confocal biomicroscopy were used for clinical follow-up every week for 4 weeks. At 4 weeks, mice were euthanized and eyes were collected at each time point for biochemical analysis using TUNEL assay, immunohistochemistry, western blot and transmission electron microscopy.

The WT and *hevin* ^*-/-*^ mice were divided into three groups. Group I animals served as naïve without any treatment. Group II had epithelial debridement with IrrPTK surgery. Each group had four time points at 1, 2, 3 and 4 weeks with 6-10 eyes at each time point. Following IrrPTK surgery, Group III animals received 10μl of 10 mg/ml of rhHevin protein (R&D Systems, Minneapolis, MN) topically at 24-hour intervals for 3 days. The experiment was terminated after 4 weeks. The mice were monitored clinically using slit lamp biomicroscopy and confocal microscopy and tissue samples were collected and analyzed as outlined in Figure 1. 

### Irregular Phototherapeutic Keratectomy (IrrPTK) Surgery

IrrPTK surgery with the high energy excimer laser was used as a model of the corneal wound healing as described earlier [35,36]. In brief, epithelial debridement was achieved using a #64 surgical blade (BD, Franklin Lakes, NJ) without any injury to the limbus followed by the application of irregular phototherapeutic keratectomy (IrrPTK) using a 3mm ablation zone on the central stroma at an ablation depth of 10μm using the Technolas 217z excimer laser (Bausch and Lomb, Munich, Germany). Irregular PTK (IrrPTK) was performed by placing a fine mesh screen in the path of the laser after firing 50% of the pulses to induce irregularity in the corneal stroma. 

### Slit Lamp Biomicroscopy

The severity of corneal opacity/ haze was clinically examined and photographed using slit lamp microscopy (Nikon FS-3V; NIKON, Tokyo, Japan) after 1, 2, 3 and 4 weeks post-IrrPTK surgery as described earlier [37]. 

### Confocal Microscopy

In vivo confocal microscopy was performed postoperatively at 1, 2, 3 and 4 weeks using a scanning confocal microscope (HRT3; Heidelberg Engineering GmbH, Heidelberg, Germany) as previously described [37]. Briefly, an immersion fluid, carbomer gel (Vidisic, Mann Pharma, Berlin, Germany) was used to prevent direct contact between the corneal surface and the objective lens. Central cornea region of each mouse was examined with 2 z-axis scans from the epithelium to the endothelium. The post-surgery corneas were assessed at the level of reflectivity in the anterior stromal layer with infiltration of inflammatory cells and activated keratocytes.

### TUNEL Assay

Apoptotic cells were analyzed in post-IrrPTK-treated WT and *hevin* ^*-/-*^ mice corneas using the in situ cell death detection kit as per manufacturer’s instructions (Roche Applied Science, Indianapolis, IN) and as described earlier [38]. Briefly, the eyes were collected and embedded in OCT (Sakura FineTek, Torrance, CA) and 6 μm thick sections were cut using a Microm HM550 cryostat (Microm, Walldorf, Germany). The enzyme was added to the samples and slides were then mounted with 4',6-diamidino-2-phenylindole (DAPI; Vector labs, Bulingame, CA) for nuclear staining. Sections were observed and imaged with Zeiss Axioplan 2 fluorescence microscope (Carl Zeiss, Oberkochen, Germany) using magnifications of 200X.

### Immunostaining

For immunohistochemistry and immunofluorescence studies, the whole eye was removed and embedded in OCT freezing compound (Sakura FineTek) within a 15mm x 15mm x 5mm mold (Fisher Scientific, Houston, TX) and stored at -80°C. Central corneal sections (6 μm thick) were cut with a cryostat (Micron HM550 GmbH). These sections were then placed on charged microscope slides (Superfrost Plus, Fisher Scientific Inc., Pittsburgh, PA) and stored at -80°C until immunostaining was performed [11]. Briefly, sections were fixed in freshly prepared cold 4% paraformaldehyde for 30 min, washed three times with 1X PBS (1st Base, Singapore), blocked with 5% BSA (Sigma-Aldrich Inc., St. Louis, MO) in 1X PBS containing 0.1% Triton X-100 (Bio-Rad Laboratories, Hercules, CA) for 1 h, and then incubated with primary antibodies diluted in the blocking solution for 2 hours at room temperature or 4°C overnight (Please see Table S1). After washing three times with 1X PBS, the sections were incubated with Alexa Fluor 594- and Alexa Fluor 488-conjugated secondary antibodies diluted to 1:800 in 1X PBS at room temperature for 1 h. After washing three times with 1X PBS, slides were mounted with DAPI (Vectashield, Vector labs). For negative controls, non-immune blocking serum was used in place of the specific primary antibody. Sections were observed and imaged with a Carl Zeiss Axioplan Z1 fluorescence microscope (Carl Zeiss).

### Western Blot

The eyes were removed with forceps and the cornea was excised with sharp scissors. For western blot, the excised corneas were snap frozen in liquid nitrogen and subsequently stored at -80°C. Total protein was extracted from each cornea by homogenizing it with a 5mm tungsten bead (Qiagen, Valencia, CA) in RIPA lysis buffer (Sigma) with protease inhibitor cocktail (Sigma) using the TissueLyzer (Qiagen). Samples were then centrifuged (10,000 x g) for 15 min at 4°C. Protein quantification was done by BCA protein assay (Pierce Biotechnology, Rockford, IL).

SDS loading buffer (Bio-Rad Laboratories, Hercules, CA) was added to each sample, boiled and 15-20 µg protein was loaded on to a 4-20% precast gel (Bio-Rad). After transferring to a nitrocellulose membrane, blots were blocked in 1X PBS containing 5% non-fat milk (Bio-Rad) followed by overnight incubation with the primary antibody at 4°C (Please see Table S1). The membranes were then washed vigorously three times each for 5 min in 1X PBS and 0.1% Tween-20. The HRP-conjugated secondary antibody was then applied at a dilution of 1:10,000 and the blots were incubated for 1 hour at room temperature. Immunoreactivity was visualized with Super Signal West Femto chemiluminescence reagent (Pierce).

### In situ zymography

In situ zymography was performed on mice corneas to localize the global MMPs activity during corneal wound healing using gelatinase/collagenase assay kit (EnzChek, Invitrogen, Carlsbad, CA). Briefly, sections were incubated at room temperature for 2 hours with reaction buffer (0.05 M TrisHCl, 0.15 M NaCl, 5 mM CaCl2, and 0.2 mM NaN3, pH 7.6) containing 40 mg/ml FITC-labeled DQ gelatin.1,10-phenanthroline (50 mM), a MMP inhibitor, was added to the frozen sections as a negative control, before applying the FITC-conjugated DQ gelatin. Sections were incubated and washed three times with 1X PBS for 5 min and counterstained with DAPI (Vector labs). The gelatinolytic activity of MMPs was analyzed and imaged using Zeiss Axio Imager Z1 fluorescence microscope with ApoTome system (Zeiss). 

### Electron Microscopy

The mouse eyes were collected and immediately fixed in cold 2.0% glutaraldehyde, 2% paraformaldehyde, and 0.1 M sodium cacodylate, pH 7.4 (Electron Microscopy Sciences, Washington, PA) overnight at 4°C. The tissues were then washed in sodium cacodylate buffer and rinsed with distilled water. For transmission electron microscopy (TEM), the samples were post-fixed in 1% osmium tetra-oxide and potassium ferrocyanide. After extensive rinsing with the sodium cacodylate buffer, tissues were dehydrated in a graded series of ethanol, and embedded in Araldite (Electron Microscopy Sciences). The ultra-thin sections of 60–80 nm were collected on copper grids, doubled-stained with uranyl acetate and lead citrate for 20 min each, then viewed and photographed using a Philips EM 208S Transmission Electron Microscope (FEI Electron Optics BV, Eindoven, The Netherlands) at 100kv as described earlier [37]. For light microscopy, semi-thin sections of 0.5–1µm thickness were cut with a Reichert-Jung Ultracut E Ultramicrotome (C. Reichert Optische Werke AG, Vienna, Austria), counter-stained with toluidine blue/basic fuchsin stain, and examined using an Axioplan Zeiss Light Microscope (Carl Zeiss).

### Statistical Analysis

Data was expressed as mean ± standard error (SE). The significance of differences among groups was determined by the two-tailed Student's t-test and one-way analysis of variance with Newman-Keuls post-hoc analysis using GraphPad Prism 6.0, where applicable. The data with P<0.05 was considered significant.

## Results

### Hevin is expressed in injured cornea during early stages of wound healing

**Figure 2:**
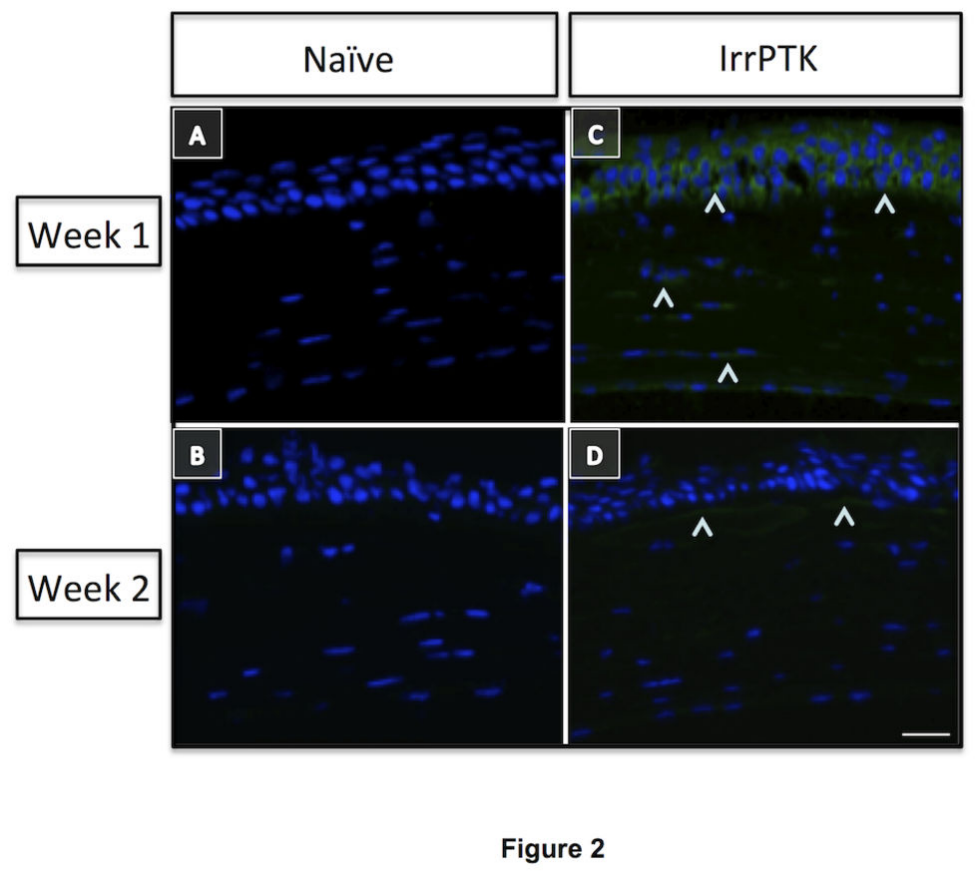
Hevin is upregulated during corneal wound healing. Hevin immunostaining (^) in WT mice showed minimal expression in naïve eyes at week 1 and 2 (A, B). IrrPTK-surgery to these mice corneas exhibited upregulation of Hevin expression in 1 week post-surgery (C), which was reduced in week 2 (D), suggesting involvement of Hevin in the early stages of tissue remodeling during corneal wound healing. Scale bar = 25μm.

Post-IrrPTK-treated WT mouse corneas exhibit hevin expression in epithelial and stromal fibroblast cells at week 1 and 2 (Figure 2C-D) as observed by immunohistochemistry. We found that hevin was not expressed in naïve (Figure 2A-B) or IrrPTK-treated at week 3 and 4 (data not shown). These results suggest that hevin is actively engaged in mouse corneal tissue remodeling at the early stages of corneal wound healing.

### Hevin null mice exhibit TUNEL-positive apoptotic cells in the corneal stroma

**Figure 3:**
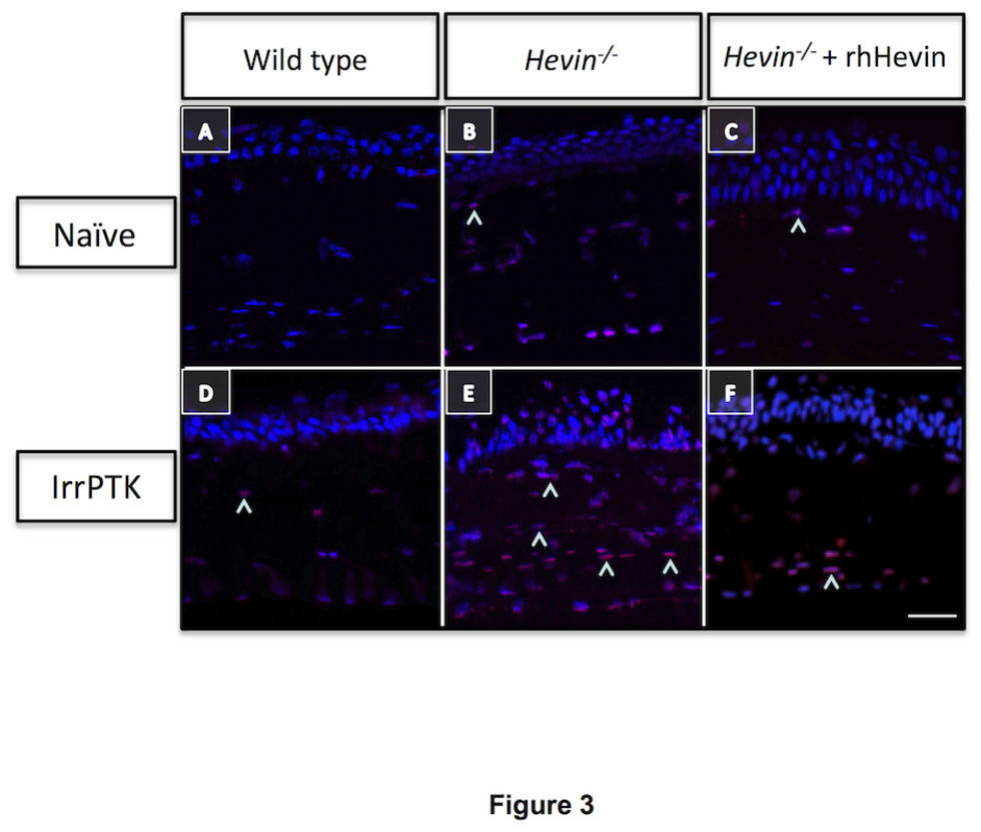
*Hevin^-/-^* mice exhibit excessive cell death in the corneal stroma after IrrPTK surgery. WT naïve mouse showed no TUNEL+ve cells in the corneal stroma (A), whereas few apoptotic cells (^) were seen in naïve state of *Hevin^-/-^* mice (B,C). IrrPTK-treatment significantly increased cell death in *Hevin^-/-^* corneal stroma (E) compared to the fewer TUNEL+ve cells observed in WT stroma (D). rhHevin prevented these cells from undergoing cell death after IrrPTK (F). Scale bar = 25μm.

We observed that *hevin* ^*-/-*^ mice corneas exhibit few apoptotic cells in the naïve state (Figure 3B) compared to their WT counterpart (Figure 3A). IrrPTK-surgery results in massive cell death in the hevin null mouse cornea at week 4 (Figure 3E). On the contrary, a few apoptotic cells were seen in 4 weeks treated WT corneas (Figure 3D) as expected due to the excimer ablation. Exogenous application of rhHevin to these corneas protected cells from undergoing apoptotic cell death in both groups (Figure 3C and 3F). 

### Hevin^-/-^ mice prolonged the inflammatory state of corneal fibroblasts

**Figure 4:**
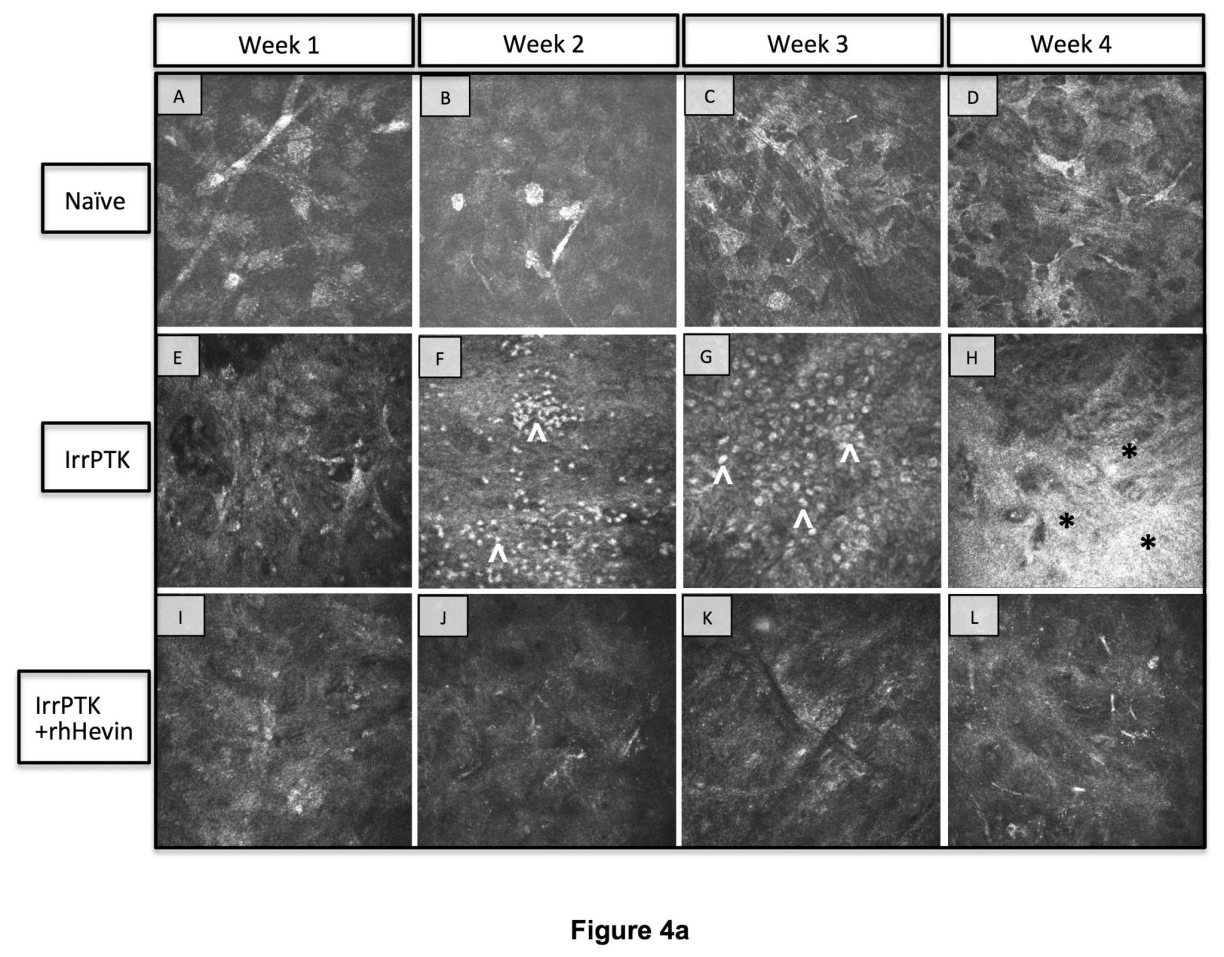
In vivo confocal microscopy in WT and *Hevin^-/-^* mouse. Naïve WT (A-D) and *Hevin^-/-^* (A’-D’) mice corneas served as control samples and exhibit no cell infiltrates and stromal haze. IrrPTK-surgery triggered infiltration of inflammatory cells (^) at 2 weeks post-op in WT mice (F), which subsides by week 4 (H). These inflammatory events are followed by the development of corneal haze (*) as seen in week 4 samples (H). In *Hevin^-/-^* mouse, inflammatory cells are seen as early as week 1 post-op (E’), which continued to increase up to week 4 (F’-H’). Supplementation of exogenous rhHevin decreased cell infiltration and impeded stromal haze formation in WT (I-L) and *Hevin^-/-^* mice (I’-L’), completely eliminated inflammatory cells in WT corneas (I-L) though low numbers of inflammatory cells were still observed in *Hevin^-/-^* mouse (I’-L’).

The inflammatory index in the mouse cornea was assessed clinically by in vivo confocal microscopy and biochemically by CD11b expression, a monocyte marker expressed in the corneal stroma after injury [39]. Infiltrating inflammatory cells start to appear as early as week 1 in WT (Figure 4A) and *hevin* ^*-/-*^ corneas (Figure 4A’), with maximal expression seen at 2-3 weeks in WT (Figure 4F-G) which subsided at week 4 (Figure 4H). The most remarkable observation was made in the *hevin* ^*-/-*^ mice, where a continual infiltration of inflammatory cells was observed in the anterior stroma after injury (Figures 4F’-H’). These results indicate that *hevin* ^*-/-*^ corneas showed increased infiltrating cells and drive stromal cells to a chronic inflammation state. On the contrary, topical application of rhHevin to *hevin* ^*-/-*^ corneas decreased cell infiltration and reduced inflammation (Figure 4I’-L’) as compared to the untreated group (Figure 4A’-D’). Similarly, WT mouse corneas showed improved recovery in terms of inflammation (Figure 4I-L) after treatment with rhHevin. 

**Figure 5:**
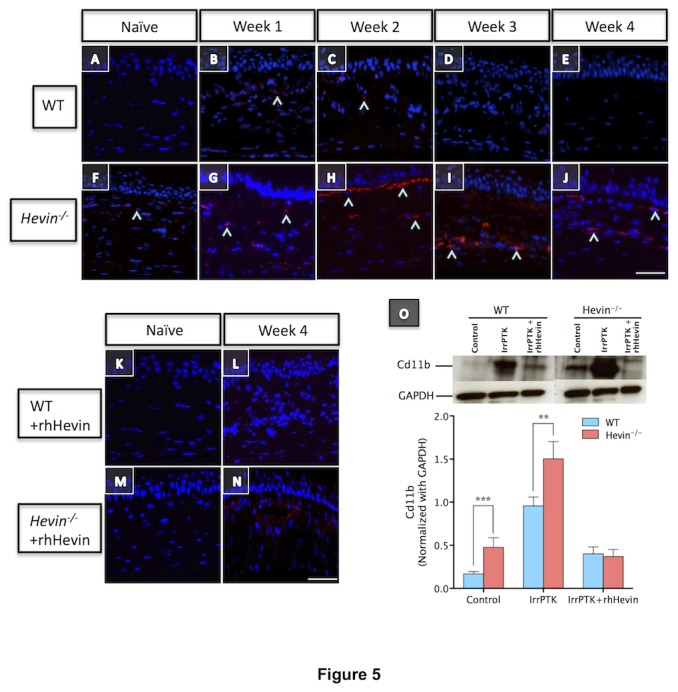
*Hevin^-/-^* mice prolonged the inflammatory state of the corneal fibroblasts. WT mouse cornea showed fewer CD11b in weeks 1 and 2 following IrrPTK (B,C), which reduced progressively in week 3 and 4 (D,E) during wound healing. *Hevin^-/-^* mice showed significantly large number of CD11b+ cells in the naïve state (F), with persistent increase over 4-weeks post-op (G-J). Application of rhHevin (K-N) reduced CD11b+ve inflammatory in *Hevin^-/-^* mice by week 4 (N). Western blot analysis showed ~2-fold increase in CD11b expression in *Hevin^-/-^* mice compared to the naïve WT corneas and further increase at 4 weeks after IrrPTK surgery, which can be reduced to near normal levels after rhHevin treatment (O). ***, P<0.001. Scale bar = 25μm.

These results were confirmed by immunohistochemistry where the *hevin* ^*-/-*^ mouse showed infiltrating CD11b+ cells in the naïve tissue indicating inflamed corneas (Figure 5F). IrrPTK-treatment to these mice exaggerated CD11b+ inflammatory cells in the corneal stroma throughout the experimental time period (Figure 5G-J). WT corneas showed a similar progressive increase in the expression of CD11b up to 3 weeks (Figure 5B-D), which was then reduced in the later stages of wound healing (Figure 5E). These inflammatory events can be rescued by the topical application of rhHevin as demonstrated by immunohistochemistry (Figure 5K-N). Western blot analysis further supported these results indicating that the loss of hevin results in ~2-fold increase in the CD11b expression compared to the naïve WT mice (P<0.001). It is interesting to note that 4 weeks IrrPTK-treated WT and hevin^-/-^ corneas showed no significant differences in the CD11b expression in western blot analysis (Figure 5O). Exogenous application of rhHevin resulted in decreased inflammatory status in these mice and showed significant decrease in the Cd11b expression in post-IrrPTK treated WT (P<0.001) and *hevin* ^*-/-*^ (P<0.001) mice compared to the IrrPTK group (Figure 5O). These results support our hypothesis that *hevin* ^*-/-*^ corneas are highly vulnerable to injury and exhibited prolonged inflammation that can be rescued by the addition of the exogenous rhHevin in both groups. 

### Hevin deficiency accelerates the appearance of corneal haze in the IrrPTK-treated mice

**Figure 6:**
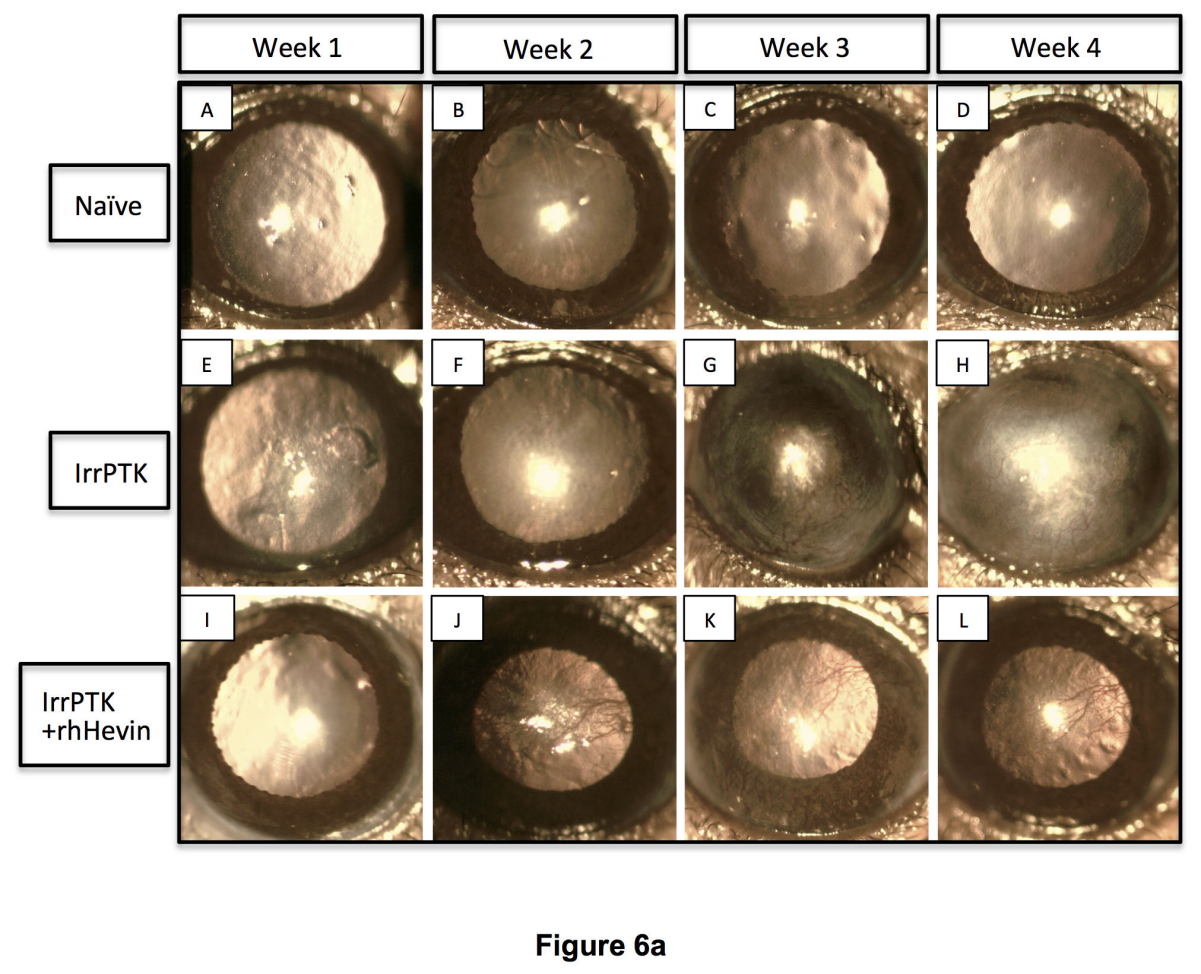
In vivo slit lamp biomicroscopy in WT and *Hevin^-/-^* mouse. Naïve WT (A-D) and *Hevin^-/-^* (A’-D’) mice corneas served as control without any treatment. IrrPTK-surgery in WT mouse developed corneal haze from Week 2 (F) but more prominently seen in weeks 3-4 (G-H). In *Hevin^-/-^* mouse, IrrPTK-treatment began to develop haze as early as Week 1 (E’), with increasing severity from Weeks 2-4 (F’-H’). In both WT and *hevin* ^*-/-*^, addition of exogenous rhHevin significantly reduced corneal haze and neovascularization in the zone of laser ablation (I-L; I’-L’).

Clinical evaluation of the corneal haze was performed in the WT and *hevin* ^*-/-*^ corneas using slit lamp biomicroscopy. All the corneas were completely re-epithelized within 48-72 hours after IrrPTK in both groups. The progressive accumulation of activated keratocytes near the excimer ablation site was observed as clinical haze during the post-op week 1, 2, 3 and 4 (Figure 6). The most severe corneal haze was noticed in *hevin* ^*-/-*^ corneas around 2-4 weeks (Figure 6F’-H’), and was seen as early as 1 week post-operatively in the area of laser photoablation (Figure 6E’). WT mouse cornea followed similar pattern but showed relatively low haze (Figure 6E-H). In addition, WT mice showed late corneal haze (3-4 weeks) as evident from the decreased cloudiness compared to the *hevin* ^*-/-*^ mice (Figure 6E-H vs. 6E’-H’). Exogenous application of rhHevin daily for 3 days until complete re-epithelialization resulted in early recovery of post-IrrPTK treated *hevin* ^*-/-*^ corneas (Figure 6I’-L’). Similar observations were made in WT mice (Figure 6I-L) also followed similar pattern of wound repair with decrease in corneal haze.

Corneal haze developing after IrrPTK surgery is usually accompanied by changes in stromal ECM and the appearance of infiltrating inflammatory cells, along with highly reflective particles – a characteristic feature of the activated keratocytes (myofibroblasts). This can be determined by backscattered light from the anterior stroma using confocal microscopy [37]. In the present study, *hevin* ^*-/-*^ corneas at week 1 after surgery showed more light-scattering reflective particles (Figure 4E’) in the anterior stroma compared to their respective WT corneas (Figure 4E). Over the next three weeks, increased intense reflective particles were observed in *hevin* ^*-/-*^ corneas (Figure 4F’-H’) whereas in WT mouse corneas showed high amounts of reflectivity in week 4 (Figure 4H).

### Hevin null mice exaggerates fibrosis in corneal stroma

**Figure 7:**
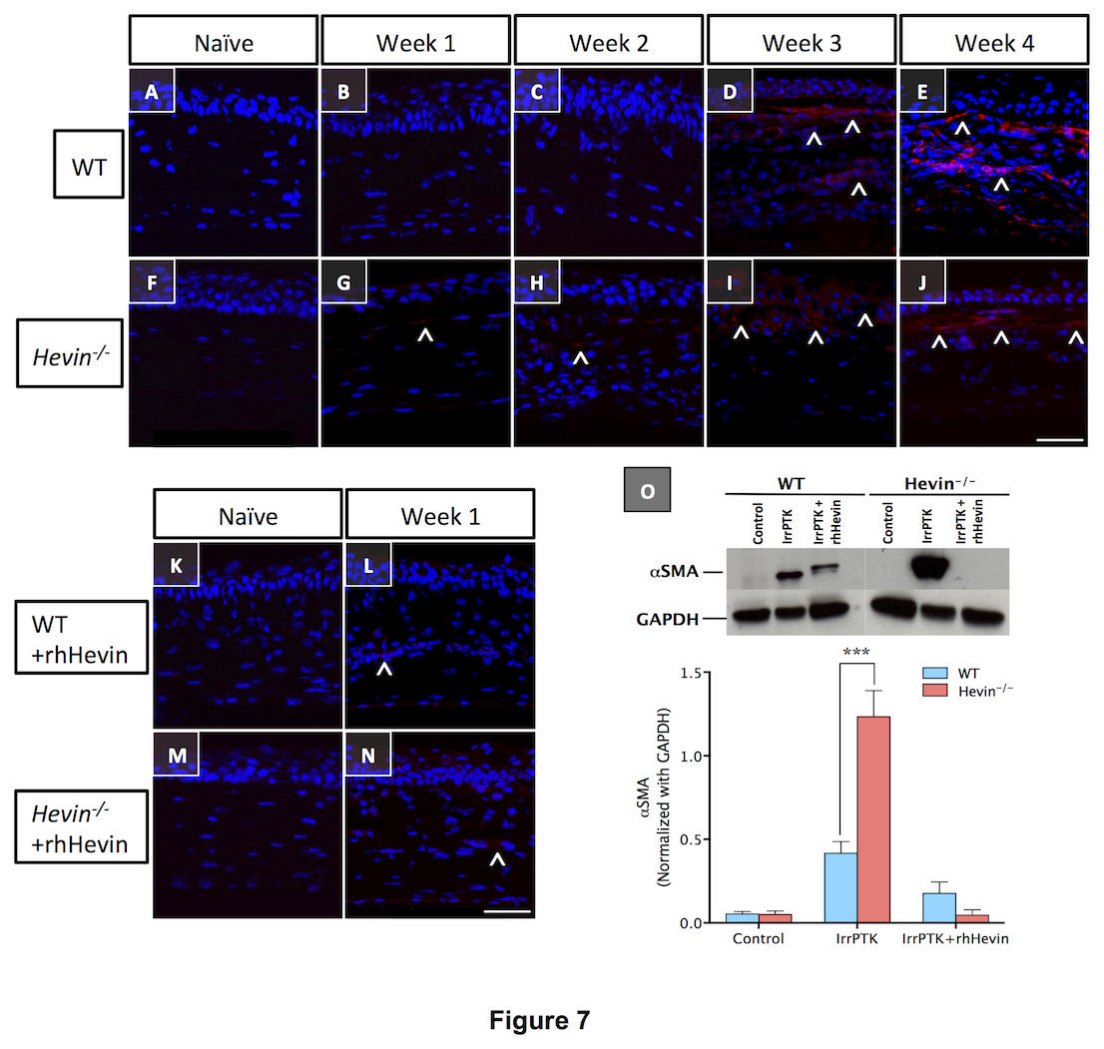
Hevin deletion leads to early stage fibrosis in mouse cornea. Activated keratocytes produced during the corneal wound healing results in the corneal fibrosis, which can be detected using immunostaining with αSMA protein. WT and *Hevin^-/-^* naive corneas showed negative expression of αSMA protein (A,F). IrrPTK-surgery results in early development of αSMA-positive cells (^) as early as Week 1 and 2 in *Hevin^-/-^* mouse (G, H), with further increase in week 3 and 4 (I, J). On the other hand, WT mouse cornea showed nominal increase in the early stages of wound healing (B,C) but peaked in week 3 and 4 (D, E). Exogenous treatment of rhHevin to the IrrPTK tissues at week 4 significantly reduced αSMA-positive cells in both WT and *Hevin^-/-^* mice as observed by immunohistochemistry (L,N) and western blotting (O). *, P<0.001. Scale bar = 25μm.

The appearance of corneal haze in IrrPTK-treated *hevin* ^*-/-*^ or WT corneas was further quantified using immunohistochemistry staining and western blot. We used alpha smooth muscle actin (αSMA), a myofibroblast (activated keratocyte) marker, to calculate the fibrotic status of the tissue. In the present study, mice corneas treated with IrrPTK developed intense αSMA-positive cells widely distributed across the anterior corneal stroma in the excimer laser ablation zone (Figure 7A-J). This confirmed the presence of myofibroblasts and the formation of haze at around 3-4 weeks as described earlier [36]. Although WT and *hevin* ^*-/-*^ corneas exhibited similar levels of haze during late stages of wound healing, significant haze was observed in *hevin* ^*-/-*^ mice as early as 1-2 weeks corneas indicating the early onset of fibrotic events in these mice (Figure 7G-H). However, treatment of IrrPTK corneas with rhHevin reduces αSMA-positive cells in the anterior stroma and reduced fibrotic processes in 4 weeks for both groups (Figure 7K-N). Western blot analysis corroborates our immunohistochemistry results (Figure 7O). The fibrotic status of the WT and *hevin* ^*-/-*^ mice corneas showed no differences as similar levels of αSMA expression were observed in naïve conditions. Post IrrPTK-treatment at 4 weeks to the hevin null mice showed significantly high levels of αSMA compared to the WT corneas (P<0.001). These fibrotic events can be suppressed in WT as well as *hevin* ^*-/-*^ corneas via topical application of rhHevin for a period of 3 days after surgery (Figure 7O; P<0.001 in both the groups).

### Loss of hevin results in excessive accumulation of irregular ECM proteins

**Figure 8:**
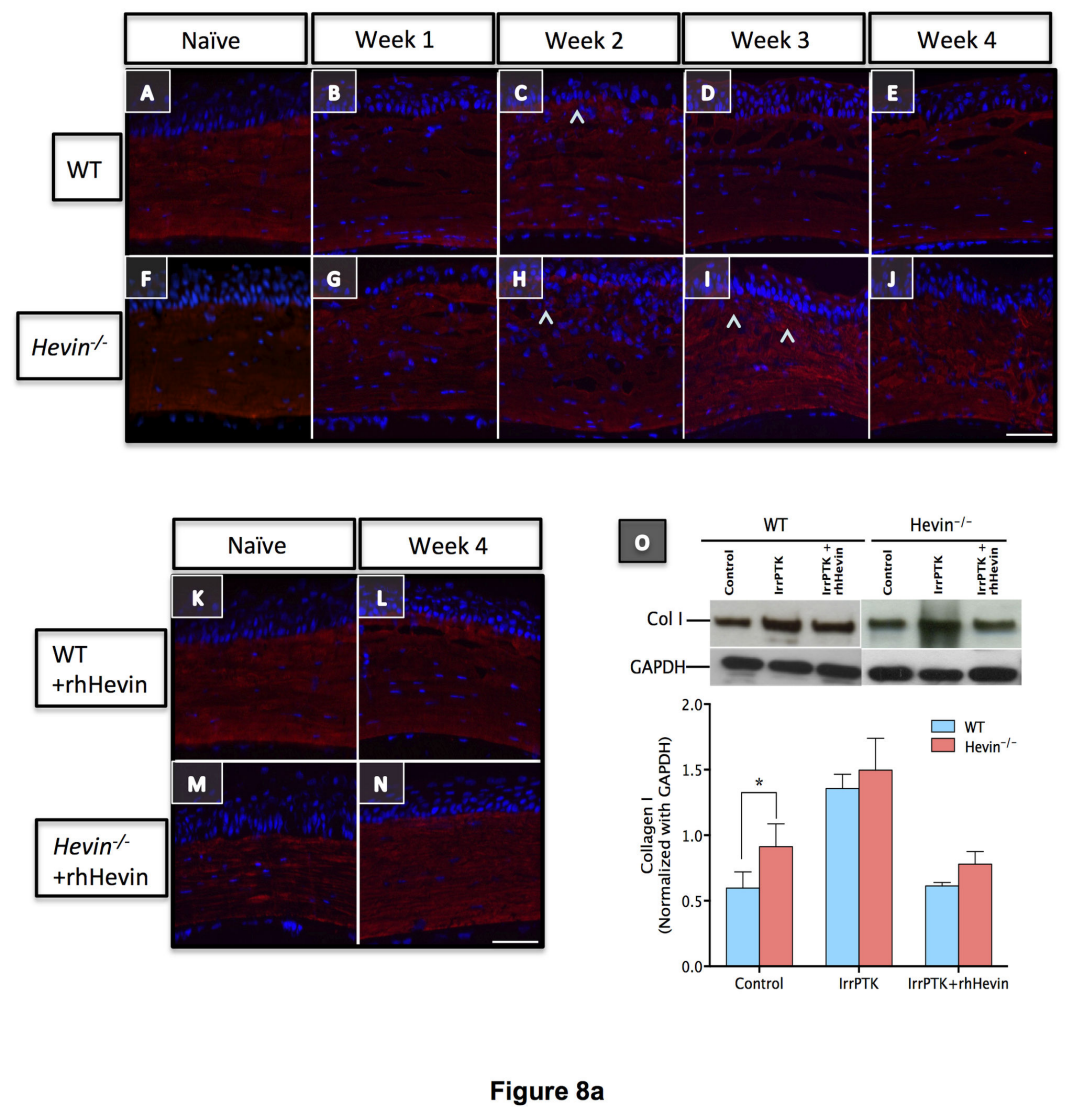
Loss of hevin results in excessive accumulation of Collagen I and Collagen IV in corneal stroma. Immunostaining of Col I showed even distribution of the ECM protein across the corneal stroma in both WT (A-E) and *Hevin^-/-^* (F-J) mouse. Marginal increase in Col I expression (^) observed in anterior stroma of IrrPTK samples (C, H, I). rhHevin treatment did not show any adverse effects on Col I staining (K-N). Western blot (O) confirm the increase of Col I expression in IrrPTK group. Col IV initially absent in naïve WT (A’) and *Hevin^-/-^* (F’) mouse showed increased and persistence expression (^) post-IrrPTK (B’-E’, G’-J’). rhHevin treatment reduced Col IV production in the WT (L’) but not in the *Hevin^-/-^* mice (N’). Similar observations were made in Western Blot analysis (O’). *, P<0.001. Scale bar = 25μm.

Myofibroblasts are known to secrete provisional ECM matrix at the injury site during the remodeling of the damaged tissue. Since collagens act as a scaffold for corneal tissue after injury, we studied the expression of collagen I (Col I) and collagen IV (Col IV) in *hevin* ^*-/-*^ mouse and compared them with WT. In naïve control, immunohistochemical staining of Col I was found to be uniformly distributed throughout the corneal stroma (Figure 8A). However, in the absence of hevin, irregular distribution of Col I was observed in untreated corneas (Figure 8F). In IrrPTK-treated groups, the intensity of Col I staining was increased in the anterior stroma without any changes to the posterior stroma (Figure 8A-J). Addition of the exogenous rhHevin to these corneas recovered the Col I expression similar to the WT controls (Figure 8K-N). In IrrPTK-treated groups, increased intensity of Col IV staining was observed throughout the corneal stroma in WT (Figure 8A’-E’) and hevin^-/-^ (Figure 8F’-J’) during the early stages of wound healing. In the physiological condition, stromal myofibroblasts upregulate the secretion of Col IV during the early events of wound healing required for the reinstatement of the basement membrane but are known to be replaced with Col I laid down by migrating keratocytes, which repopulate the injured zone. In the *hevin* ^*-/-*^ mice, we found that myofibroblasts continued to express Col IV protein in the corneal stroma thus resulting in the excess accumulation of irregular fibrotic ECM at the injury site, which is otherwise not seen in the regular ECM matrix. However, if these IrrPTK treated *hevin* ^*-/-*^ corneas were treated with exogenous rhHevin, they can be reverted to the normal WT levels with the expression of Col I (Figures 8K-N) but not Col IV (Figure 8K’-N’) in the ECM matrix. 

Western blot analysis complemented immunohistochemistry results and showed excessive accumulation of Col I (Figure 8O; P<0.001) and Col IV (Figure 8O’; P<0.001) proteins in *hevin* ^*-/-*^ mice compared to their WT counterparts after IrrPTK surgery. We found that the exogenous supplementation of rhHevin prevented accumulation of Col I (P<0.001) and Col IV (P<0.001) in WT mice and significantly reduced the expression of Col I (P<0.0001) but not Col IV in *hevin* ^*-/-*^ mice corneas (Figure 8O’).

### Loss of Hevin drives corneal stroma to the continual matrix remodeling status

**Figure 9:**
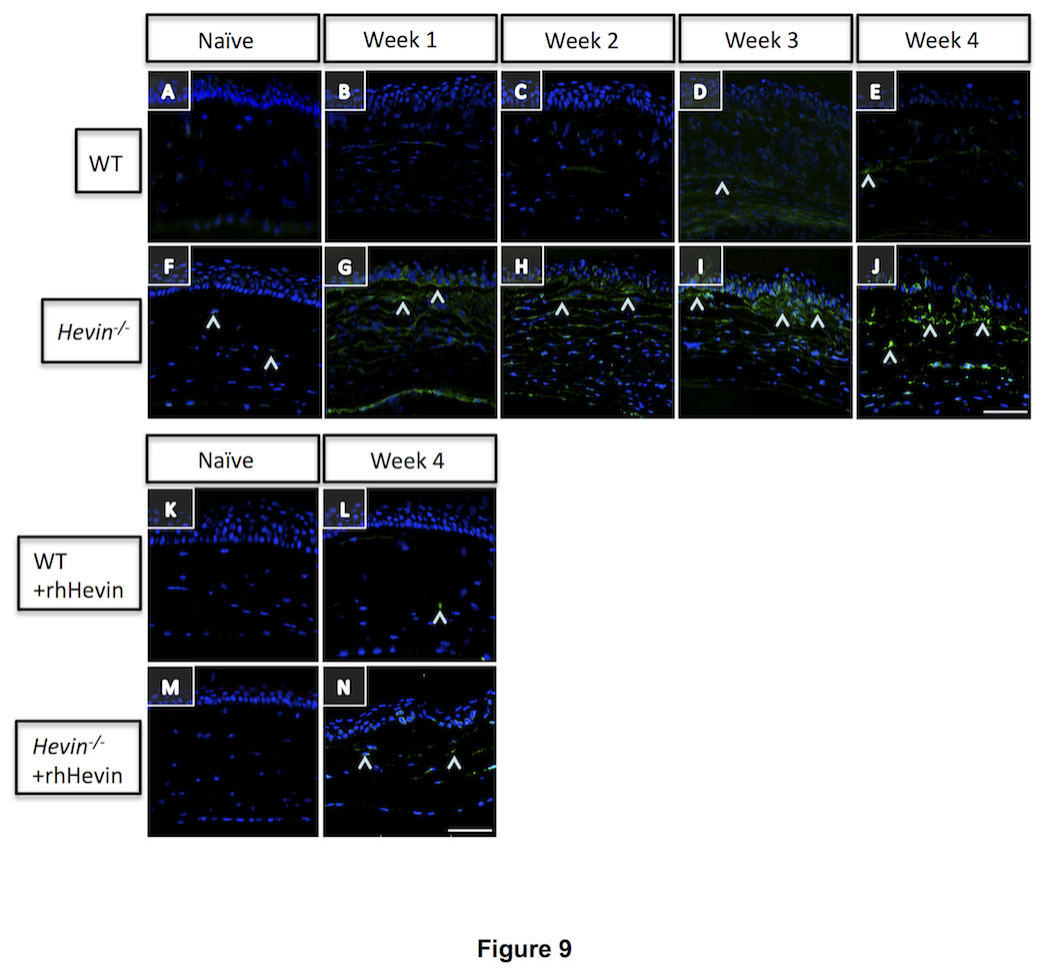
Elevated MMPs activity in *Hevin^-/-^* mouse leads to deposition of irregular ECM. Collagenase assay for MMPs activity (^) in WT (A-E) and *Hevin^-/-^* (F-J) mouse show slight increase in Week 3 and 4 post-IrrPTK in WT (D, E). Low level of MMPs activity is seen in Week 1 post-op knockout mice (G), which increases progressively from weeks 2-4 (H-J). rhHevin supplementation decreased MMPs activity to that of the naïve state L, N). Scale bar = 25μm.

There is a dynamic interrelationship between the provisional (temporary) ECM matrix deposition and matrix remodeling via activation of proteases during corneal wound healing. This is accomplished via regulation of a major class of ECM degrading enzymes called matrix metalloproteinases (MMPs). Therefore, we decided to localize the global MMPs activity in these tissues using in situ zymography. In unwounded corneas, *hevin* ^*-/-*^ mice exhibited low levels of MMPs activity (Figure 9F) in the corneal stroma compared to no activity in the WT mice corneas (Figure 9A). IrrPTK-treatment to the WT corneas showed a progressive increase in the MMPs activity in the early stages of wound repair (Figure 9B-C) and relatively stabilized in the later stages of experiments (Figure 9C-D). However, significantly higher MMPs activity was detected in the *hevin* ^*-/-*^ stroma in the first two weeks after IrrPTK (Figure 9G-H), which continued to increase throughout the experimental period (Figure 9I-J) indicating a constant remodeling state of matrix proteins. These continual matrix degradation activities can be attributed to the excessive synthesis of irregular stromal collagen in the hevin null mice (Figure 8F-J and 8F’-J’) during corneal wound healing. It is interesting to note that supplementation of rhHevin to these mice decreased the MMPs activity, comparable to the levels seen in naïve corneas in both WT (Figure 9K-L) and *hevin* ^*-/-*^ mice (Figure 9M-N), which also indicated that hevin plays a substantial role in the reorganization of the ECM protein in injured corneas. 

### Hevin gene ablation leads to increased vascularization in the cornea

IrrPTK surgery in mice results in corneal neovascularization in both WT and hevin^-/-^ mice as observed by slit lamp biomicroscopy (Figure 6). In the WT mouse cornea, neovascularization was observed in week 3 (Figure 6G) and week 4 (Figure 6H) samples, whereas it can be seen as early as week 1 in *hevin* ^*-/-*^ samples (Figure 6E’). There was a gradual increase in these vessels with time and were seen at maximum at week 4 in both groups (Figure 6H and 6H’). Supplementation of rhHevin to these mice corneas significantly reduced corneal neovascularization in WT (Figure 6I-L) and *hevin* ^*-/-*^ (Figure 6I’-L’).

**Figure 10:**
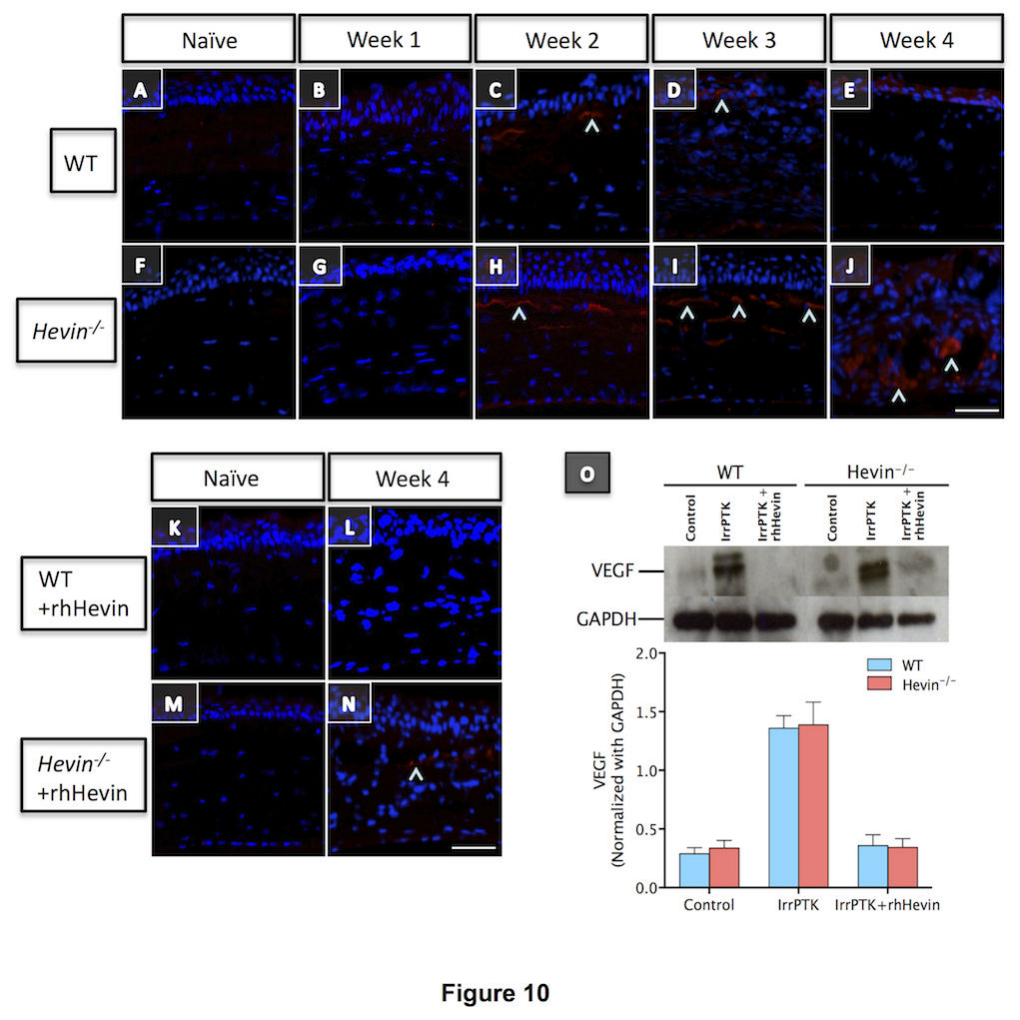
Hevin gene ablation leads to elevated VEGF expression in corneal stroma. VEGF expression (^) showed progressive increase in *Hevin^-/-^* mouse from week 2 (H), which maximizes at week 3 (I) and week 4 (J). On the contrary, WT mouse showed minimal expression of VEGF at 2 weeks post-op (C), which was further reduced in week 3 (D) and week 4 (E). rhHevin treatment reduced VEGF expression in these mice and rescued them to near normal levels at week 4 (L and N, respectively for WT and *Hevin^-/-^* corneas) as supported by western blot analysis (O). Scale bar = 25μm.

To elucidate the role of hevin in corneal neovascularization after injury, we further analyzed these injured corneas for the vascular endothelial growth factor (VEGF) expression using immunohistochemistry and western blot. In *hevin* ^*-/-*^ corneas, IrrPTK-surgery showed a progressive increase in the VEGF+ cells in the stroma (Figure 10G-J), which peaked at 4 weeks after treatment (Figure 10J). On the contrary, WT corneas showed fewer VEGF+ cells during the early stages of wound healing (Figure 10A-E). Topical application of rhHevin to the IrrPTK-treated mice lacking hevin, substantially decreased the expression of VEGF+ cells (Figure 10N). These results were further confirmed by the western blot analysis (Figure 10O). IrrPTK-injured corneas at 4 weeks showed significant increase in the expression of VEGF in both WT and *hevin* ^*-/-*^ (P<0.001 for both). Nevertheless, treatment with rhHevin reverted VEGF expression to near normal levels for both WT and *hevin* ^*-/-*^ corneas (P<0.001 for both). 

### Hevin regulates stromal cellular integrity and organization after injury

**Figure 11:**
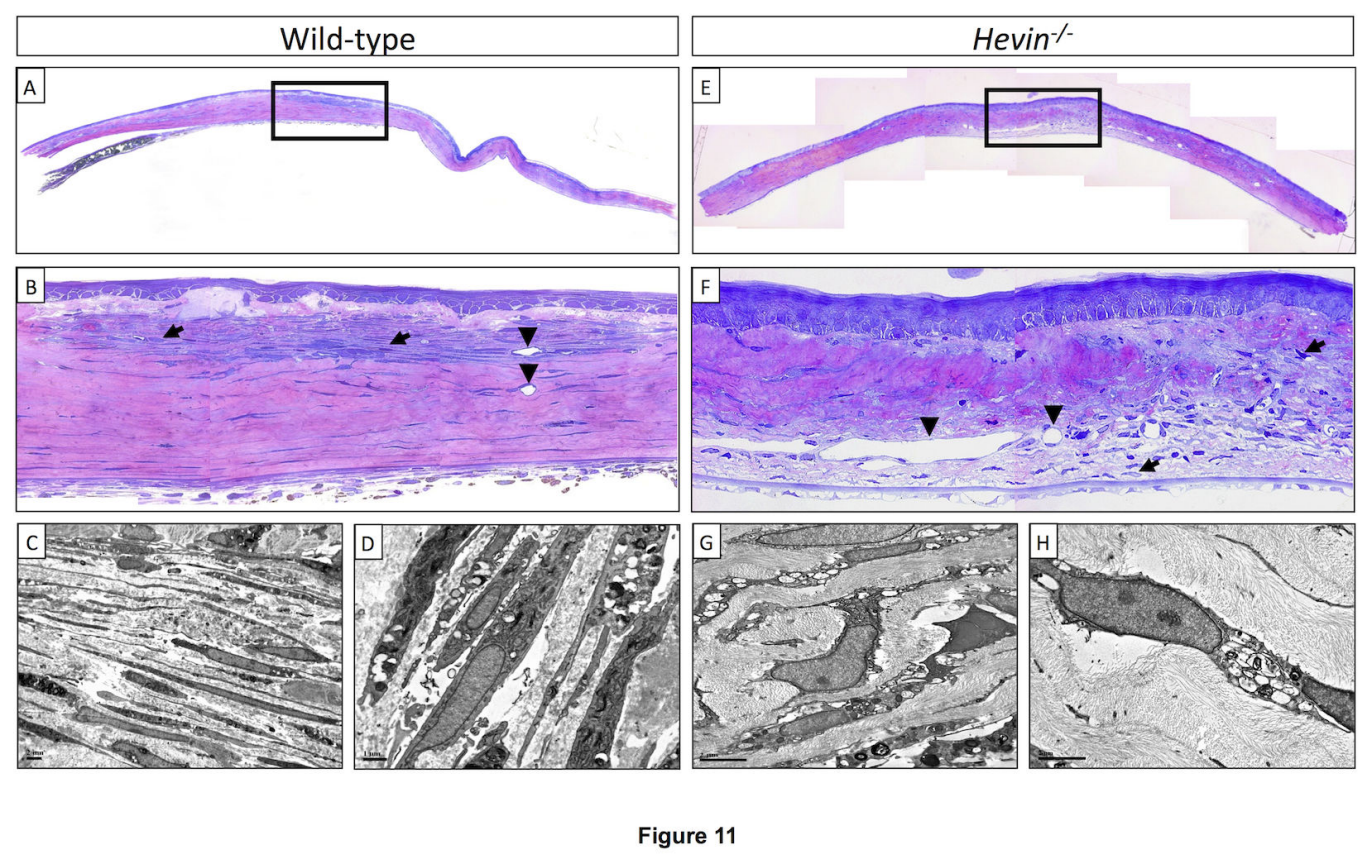
Light and Transmission electron microscopy in wild type and IrrPTK-treated corneas. *Hevin^-/-^* mice (Figure 11F) showed distinct cellular activity and epithelial cellular disorganization after IrrPTK surgery compared to the WT corneas (Figure 11B). Large vacuolated keratocytes observed in transmission electron micrographs of *Hevin^-/-^* mice (Figure 11G-H) compared to the WT corneas (Figure 11C-D).

Histology of the naïve cornea using light microscopy showed distinct cellular activity and epithelial cellular disorganization in the *hevin* ^*-/-*^ *mice* (Figure 11F) after IrrPTK surgery compared to the WT corneas (Figure 11B). Activated keratocytes repopulated and infiltrated near the injury site and were located throughout the anterior corneal stroma (Figure 11B). However, in *hevin* ^*-/-*^ group, a decrease in keratocytes density was observed in the anterior stroma (Figure 11F) where laser ablation was done as compared to the WT group (Figure 11B). Transmission electron micrographs at the IrrPTK treated corneas revealed large vacuolated keratocytes in the *hevin* ^*-/-*^ mice (Figure 11G-H) compared to the WT corneas (Figure 11C-D). We found an irregular pattern of increased stromal infiltration and activated keratocytes throughout the cornea in hevin null mouse (Figure 11G-H). 

## Discussion

Our results suggest for the first time that hevin is an integral component of the cornea and plays a pivotal role in wound healing. In order to achieve this, we used a previously established irregular phototherapeutic keratectomy (IrrPTK) mouse model of the corneal wound healing [35]. Clinical and biochemical analyses suggested that *hevin* ^*-/-*^ mouse cornea after IrrPTK surgery accelerated apoptotic cell death, chronic inflammation, severe haze, deposition of irregular extracellular matrix (ECM), and development of neovascularization in a corneal wound healing model. We found that most of these deleterious effects can be rescued by the exogenous application of rhHevin to these injured mice. 

Hevin expression in the cornea has not yet been previously studied. In the present study, we found that naïve corneas do not express hevin protein but IrrPTK-surgery corneas triggered the hevin expression during the first 2-weeks of the healing period (Figure 2). As wound healing involves an early phase of inflammatory events followed by late phases of fibrosis and remodeling of the injured tissue, the expression of hevin was no longer seen during the fibrotic stages in the 3^rd^ and 4^th^ weeks of the corneal wound healing (data not shown). Immunohistochemistry data showed that hevin was widely distributed throughout the cornea. Since IrrPTK surgery involves epithelial debridement and stromal surface ablation, hevin expression was maximally seen in the repairing epithelial cells and the anterior stroma in the first week after injury, which decreased further in week 2 and localized mainly in the anterior stroma (Figure 2). As wound healing involves an early phase of inflammatory events followed by late phases of fibrosis and remodeling of the injured tissue, the expression of hevin was no longer seen during the fibrotic stages in the 3^rd^ and 4^th^ weeks of the corneal wound healing (data not shown). Similar findings were made in studies involving skin implants where hevin was found to modulate early inflammatory events involved in dermal wounds [40]. 

Corneal keratocyte apoptosis immediately after injury has been extensively studied [13]. Modulation of this early stromal cell apoptosis is thought to be critical and rate-limiting step in an effective wound healing mechanism. Any dysregulation in the apoptotic events can lead to excessive scarring, fibrosis [41], and corneal neovascularization [42]. In clinical practice, excimer laser photorefractive keratectomy (PRK) for myopia treatment has been shown to trigger apoptotic cell death in the corneal stroma [43]. In fact, there is a direct relationship between the amount of keratocyte cell death and depth of the stromal laser ablation [44]. IrrPTK surgery in the WT cornea results in keratocyte apoptosis as early as 4-24 hours and complete recovery was achieved within a week. In contrast, mice lacking hevin exhibited a considerable number of apoptotic cells in the naïve corneal stroma. Corneal injury via IrrPTK surgery further aggravated the apoptotic events as observed by the increase in the number of TUNEL+ve cells in the week 4 corneas. However, WT corneas showed almost no apoptotic cells in the naïve or in the injured state after 4 weeks as expected. Addition of exogenous recombinant human hevin rescued keratocytes from undergoing programmed cell death. These results suggest that the hevin null mouse may have defective apoptotic machinery, which can be triggered by any injury leading to massive programmed cell death.

Clinical evaluation using in vivo confocal microscopy showed progressive accumulation of the infiltrating inflammatory cells up to 4 weeks after IrrPTK surgery in hevin deficient mice. These cells usually exist in the early phase of wound healing as seen in the WT mice (1-2 weeks) but later disappear, paving way to the repair fibroblast cells during corneal wound healing. Hevin null mice exhibited significantly higher inflammatory state compared to the WT mice as observed by the accumulation of CD11b expressing cells using immunohistochemistry and western blot analysis. A previous study by Barker et al. [40] reported that targeted disruption of the hevin gene elicits an initial acute inflammatory response in skin implants and is associated with increased biomaterial-induced inflammation. Similar observations have been made in dermal wounds where aberrant wound healing in hevin-deficient mice was attributed to its role in enhanced macrophage infiltration and accumulation around wound beds [29,34]. 

In the present study, IrrPTK-surgery in the corneal stroma resulted in the initial acute inflammatory response, including recruitment of monocyte-derived macrophages as observed by the increased expression of CD11b in the WT and hevin null mouse (Figure 5). We, and others have previously reported an increased recruitment of CD11b+ve cells in the initial phase of corneal injury [39] and diseases [45]. This early inflammatory response is prerequisite to the late stage fibrotic events accompanied by proliferation and migration of keratocytes to effectively close a wound [46,47]. We found that loss of hevin activates a chronic inflammatory state as seen by consistent CD11b expression in the later stages (third and the fourth weeks) of corneal wound healing. Supplementation of rhHevin to these mice after IrrPTK recovered them completely with no late stage inflammation development as observed by a significant drop in the CD11b expression levels. These results are mirrored in our in vivo confocal microscopy where IrrPTK surgery in hevin null mice showed progressive and excessive accumulation of infiltrating inflammatory cells in the wounded area throughout the experimental period (week 1-4). In contrast, rhHevin-treatment immediately after IrrPTK surgery substantially decreased the late stage recruitment and accumulation of inflammatory cells in WT and *hevin* ^*-/-*^ mice. These results suggest that Hevin might play a major role in maintaining the acute inflammatory index of the cornea after injury. Currently, how hevin influences the inflammatory response is not known but its suggested role in the adaptive immunity might contribute to its anti-inflammatory response [40,48]. 

Matricellular proteins are known to be involved in the regulation of tissue development, ECM deposition and assembly, anti-adhesive property, and modulation of growth factor/cytokine signaling pathways [18,48]. Hevin has been found to alter collagen fibrillogenesis in dermal wounds [34]. One of the most critical steps in corneal wound healing is its exceptional ability to reinstate the stromal tissue to its physiological quiescent state after injury [2]. This process also ensures that corneal keratocytes remain tightly packed in the ECM matrix to provide a clear and transparent tissue, a fundamental requirement for the normal vision. Clinical examination using slit lamp biomicroscopy in *hevin* ^*-/-*^ mice corneas after IrrPTK surgery revealed severe clinical corneal haze as early as 1-2 weeks after injury and continued to increase throughout the experimental duration (4 weeks). However, WT corneas exhibited haze in the late fibrotic stage of corneal wound healing at 3-4 weeks after surgery as reported previously [35]. 

In vivo confocal microscopy observations revealed a gradual increase in the density of highly reflective particles in the anterior stroma in both WT and *hevin* ^*-/-*^ groups over the period of four weeks after IrrPTK; but was significantly greater and appeared as early as 1 week after surgery in *hevin* ^*-/-*^ corneas. This was primarily due to the transformation of keratocytes into highly reflective myofibroblasts and deposition of a new provisional ECM, thus resulting in clinical haze [2,41,49]. This also indicates that loss of hevin increased myofibroblast population as early as 1 week after injury and secreted excessive irregular ECM proteins in the photoablated corneal tissue associated with an abnormal wound healing response. Conversely, post-surgical treatment of rhHevin topically to the injured corneas significantly reduced haze suggesting that hevin may play a role in the myofibroblast formation and secreted matrix remodeling. In fact, hevin-null mice have been shown to close and heal skin wounds faster characterized by enhanced macrophage infiltration around wound beds, early appearance of immature extracellular matrix, and disorganized collagen fibrils [29,34]. However, significant reduction in the intensity of highly reflective keratocytes and haze were observed in rhHevin-supplemented corneas after IrrPTK injury. 

The fibrotic events in the cornea are characterized by the expression of myofibroblasts, a repair keratocyte phenotype, expressed during corneal wound healing and can be detected using a marker, αSMA. The expression levels of αSMA are often correlated to the amount of stromal haze seen in the cornea [49]. IrrPTK surgery resulted in the upregulation of αSMA expression in the WT and *hevin* ^*-/-*^ mice corneas. We found that αSMA expression was seen as early as 1-2 weeks and significantly higher in the *hevin* ^*-/-*^ mice after injury compared to 3-4 weeks in the wild type corneas, corroborating our similar findings in the clinical analysis using slit lamp and confocal microscopy. ECM proteins such as Collagen I, which constitute a major component of the cornea stroma [50] also showed significantly higher levels in the *hevin* ^*-/-*^ mice implicating its role in the continually reorganizing matrix in these mice. The presence of collagen IV in the ablation zone, otherwise not seen in normal corneal stroma, also suggested that the myofibroblasts produced irregular ECM proteins in the *hevin* ^*-/-*^ mice. Exogenous application of rhHevin to IrrPTK injured corneas reduced αSMA and collagen I but not collagen IV expression in the hevin^-/-^ mice to control levels. These findings indicate hevin’s function in limiting excessive irregular ECM secretion in the early stages to maintain normal wound healing response but not in the later stages where collagen IV deposition was seen in the anterior stroma of the hevin null mouse. This is also supported by the fact that hevin was expressed only in the first two weeks of the corneal wound healing after IrrPTK injury (Figure 2).

An important feature of matricellular proteins such as hevin is their role in de-adhesion and cell migration to regulate endothelial cell attachment involved in new vessel formation [51]. Hevin has been detected in the vascular endothelium of several tissues and regarded as the second most abundant pan-endothelial marker in normal and tumor vascular endothelial cells [52]. In the present study, hevin null mice exhibited marginally high levels of neovascularized corneas compared to the WT counterparts as observed in the slit lamp microscopy. VEGF expression in the WT and *hevin* ^*-/-*^ mice increased after IrrPTK surgery but there were no differences in their expression levels between the two groups. This supports the notion that cornea being avascular tissue does not express hevin or VEGF in the naïve state and hence no observed differences in the physiological conditions. A previous study on skin implants also showed no substantial differences in the vessel diameters /vessel profiles of the WT and hevin null mouse [40]. Supplementation of rhHevin to corneas after IrrPTK surgery regressed the development of new vessel formation in both WT and *hevin* ^*-/-*^ mice corneas. It is interesting to note that the expression of hevin in the early inflammatory phase of wound healing might be beneficial to corneal tissue as it regulates the development of corneal neovascularization in the later stages of wound repair. TSP-1, another member of the matricellular proteins, has been suggested to play a similar anti-angiogenic role in dermal wound repair [53]. 

In summary, for the first time, this study demonstrates the expression, distribution and functional role of hevin in the cornea. We found that the hevin null mouse is highly vulnerable to injury/insult and developed chronic inflammation and early fibrotic events resulting in the modification of multiple components in corneal wound healing. Supplementation of rhHevin rescued the IrrPTK corneal injury model and hence was able to restrict early stage infiltration of inflammatory cells followed by the reduction in fibrotic events and irregular ECM deposition at the wounded area. Thus, hevin is transiently and temporally expressed at the injury site during the early phase of wound repair to prevent chronic inflammation and excessive fibrosis in the cornea.

## Supporting Information

Table S1 **List of Antibodies used in the study.**
(PDF)
