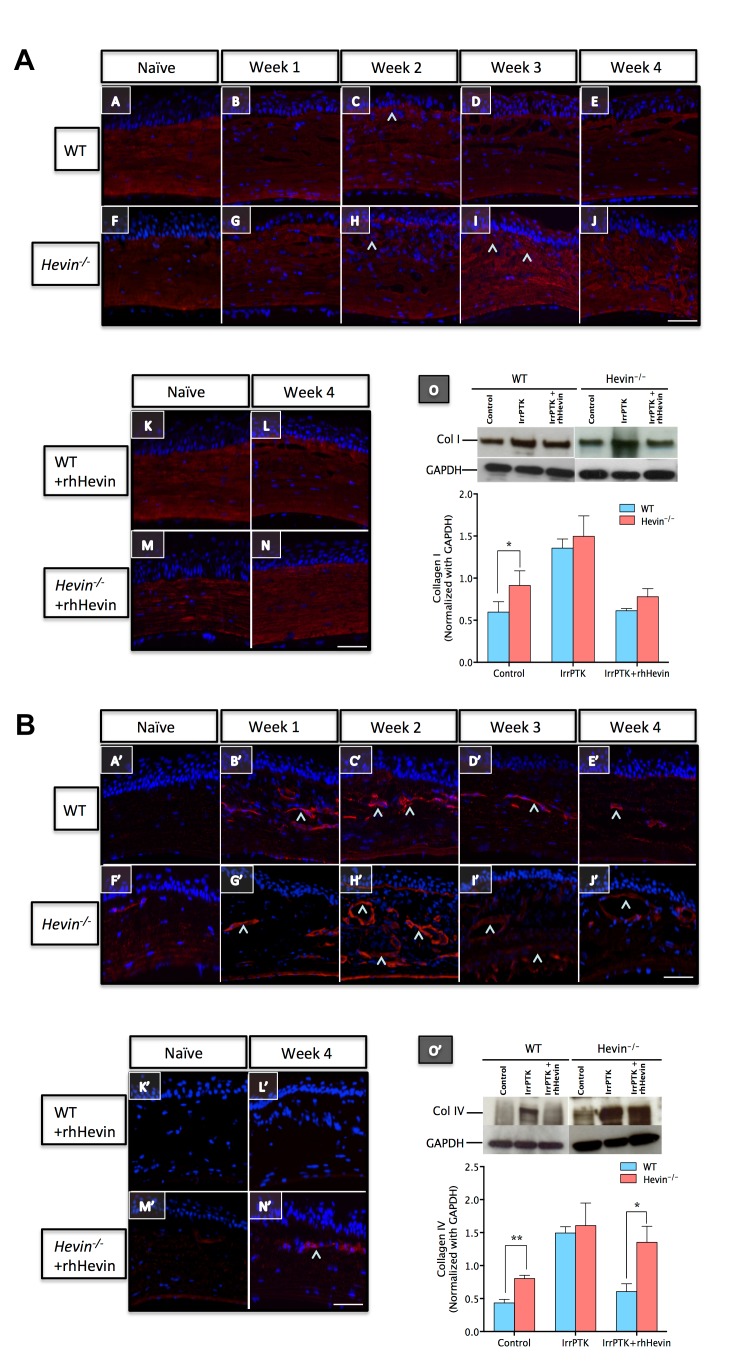# Correction: Hevin Plays a Pivotal Role in Corneal Wound Healing

**DOI:** 10.1371/annotation/03bcd6d6-0b9c-4591-a3f3-165201253234

**Published:** 2014-01-02

**Authors:** Shyam S. Chaurasia, Promoda R. Perera, Rebekah Poh, Rayne R. Lim, Tina T. Wong, Jodhbir S. Mehta

Figures 4b, 6b, and 8b are missing from the manuscript. Please see the complete Figures 4, 6, and 8 here:

Figure 4: 

**Figure:**
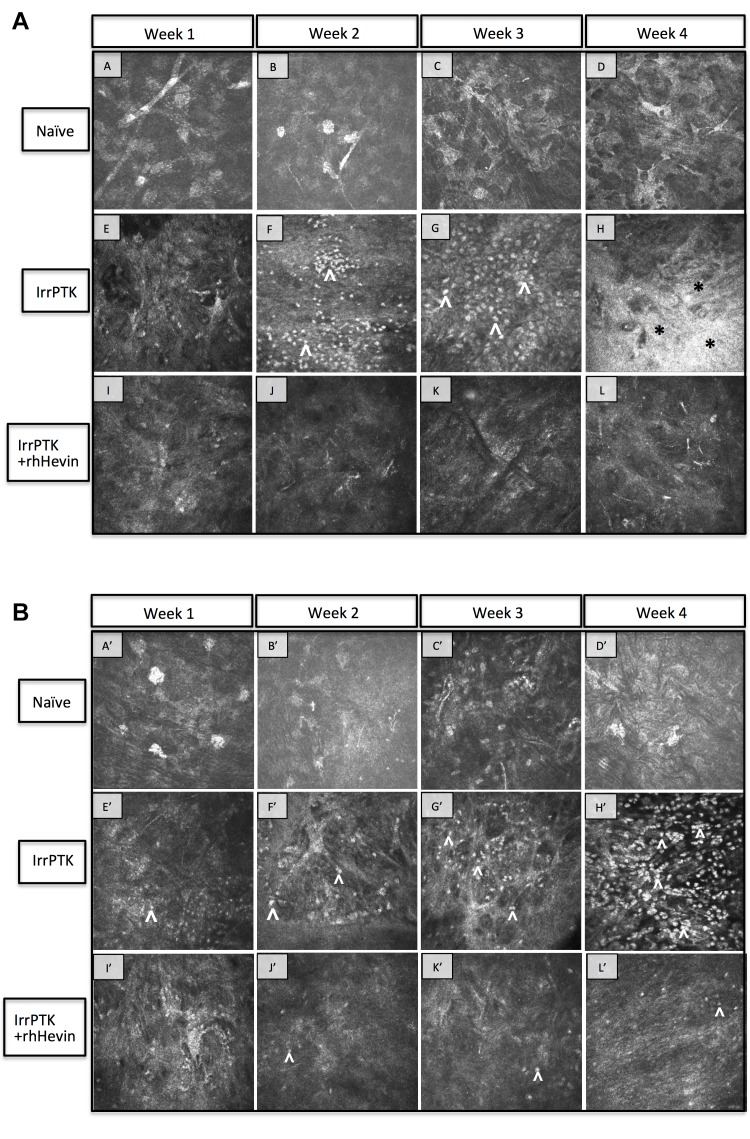


Figure 6: 

**Figure:**
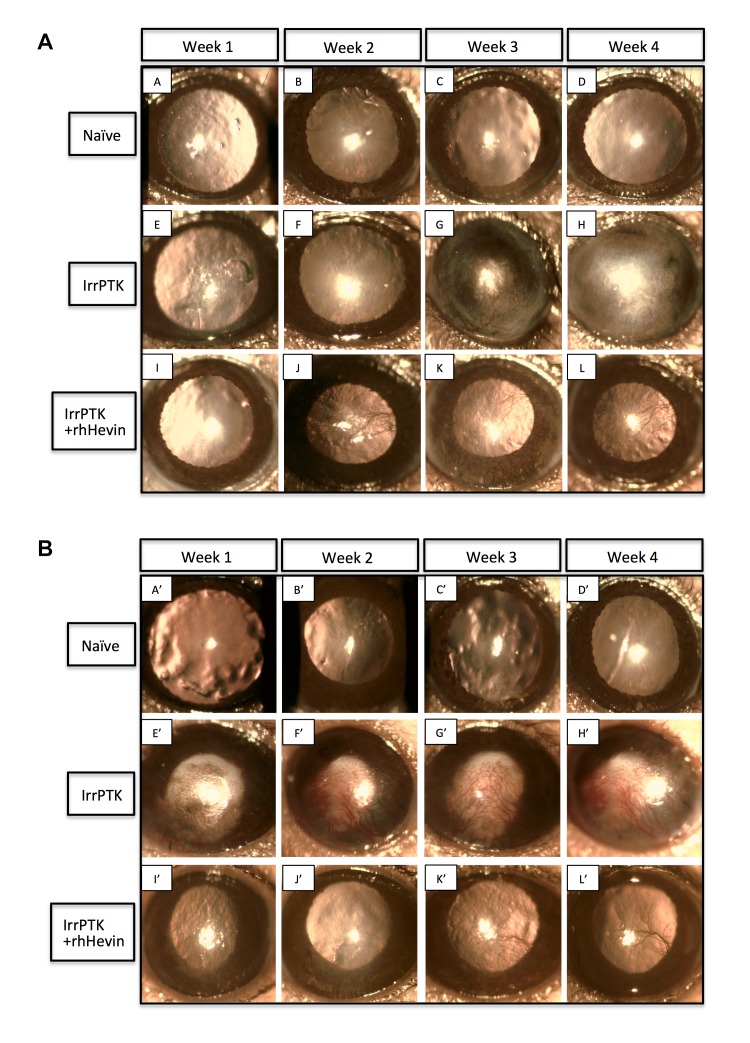


Figure 8: 

**Figure:**