# Supplementary material for: Hevin Plays a Pivotal Role in Corneal Wound Healing
Source: PLoS One. 2013 Nov 26;8(11):e81544. doi: 10.1371/journal.pone.0081544 (PMC3841198; doi:10.1371/journal.pone.0081544)
Supplement: Table S1 — List of Antibodies used in the study. (PDF) [file pone.0081544.s001.pdf]

Table S1. List of Antibodies used in the study.

Primary Antibody	Type	Dilution Factor	
		IHC-Fr	WB
αSMA	Rabbit monoclonal	1:50	1:100
CD11b	Rat monoclonal	1:100	1:200
VEGF	Rabbit polyclonal	1:100	1:100
Hevin	Mouse monoclonal	1:50	NA
Collagen 1	Rabbit polyclonal	1:100	1:100
Collagen iv	Rabbit polyclonal	1:100	1:100
